# Recent advances in branching mechanisms underlying neuronal morphogenesis

**DOI:** 10.12688/f1000research.16038.1

**Published:** 2018-11-12

**Authors:** Shalini Menon, Stephanie Gupton

**Affiliations:** 1 Department of Cell Biology and Physiology, The University of North Carolina at Chapel Hill, Chapel Hill, Chapel Hill, NC, 27599, USA; 2 Neuroscience Center, The University of North Carolina at Chapel Hill, Chapel Hill, NC, 27599, USA; 3 Lineberger Comprehensive Cancer Center, University of North Carolina at Chapel Hill, Chapel Hill, NC, 27599, USA

**Keywords:** actin, microtubules, exocytosis, branching, arborization, gene expression, activity

## Abstract

Proper neuronal wiring is central to all bodily functions, sensory perception, cognition, memory, and learning. Establishment of a functional neuronal circuit is a highly regulated and dynamic process involving axonal and dendritic branching and navigation toward appropriate targets and connection partners. This intricate circuitry includes axo-dendritic synapse formation, synaptic connections formed with effector cells, and extensive dendritic arborization that function to receive and transmit mechanical and chemical sensory inputs. Such complexity is primarily achieved by extensive axonal and dendritic branch formation and pruning. Fundamental to neuronal branching are cytoskeletal dynamics and plasma membrane expansion, both of which are regulated via numerous extracellular and intracellular signaling mechanisms and molecules. This review focuses on recent advances in understanding the biology of neuronal branching.

## Introduction

Elaborate branched structures are present across fungi, plant, and animal kingdoms. These beautiful forms are paramount to function, including mycelial colonization, plant vascularization, and the physiological functions of the circulatory, respiratory, renal, and nervous systems. The unifying consequence of branching is maximization of surface area, used for self-propagation, efficient exchange of gases and fluids, or storing and relaying of information. Experimental science, mathematical studies, and computational modeling have revealed numerous mechanisms and underlying principles of branching morphogenesis in organisms, organs, and single cells such as neurons. General reviews on branching morphogenesis have been recently published ^1– 4^. Here we focus on recent advances in neuronal branching morphogenesis.

Mature neuronal circuitry comprises an astounding density of synapses between axons and dendrites or between neurons and effector cells. The capacity of a single axon to form multiple synapses is facilitated by axonal branching, which occurs via bifurcation of the tip of the extending axon or collateral branching from the axon shaft. Dendrite arborization increases synaptic capacity but is also essential for sensory perception. Dendritic arborization patterns are strikingly different between neuronal types and are dictated by the number and types of synaptic or sensory inputs received and the geometry and size of the receptive fields. The extensively branched phenotypes of neurons were elaborately depicted by Santiago Ramón y Cajal in the 19th century and have since been an area of extensive research. Failure to establish proper branching patterns and circuitry leads to various neurodevelopmental and neuropsychiatric disorders ^5– 10^.

**Figure 1.**
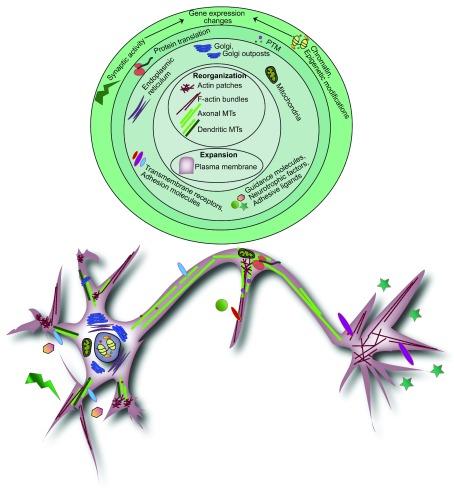
Hierarchical events of neuronal branching. Neuronal branching is a coordinated process that requires extensive filamentous actin and microtubule (MT) reorganization and plasma membrane expansion, depicted in the center of the branching hierarchy. These foundational events are spatially and temporally regulated by numerous signaling and mechanical pathways in a coordinated fashion. These pathways are initiated in response to synaptic activity, extracellular guidance molecules, neurotrophic factors, or adhesive ligands. This outside-in signaling results in extensive cytoskeletal and membrane changes regulated directly through signal transduction, transport and sorting of organelles and cargo-carrying vesicles, and energy production or indirectly through gene expression changes via transcriptional regulation, epigenetic modifications, post-transcriptional and post-translational modifications (PTMs), and local protein synthesis.

Branching morphogenesis is a “Herculean task”, which at its foundation involves dramatic plasma membrane expansion and dynamic cytoskeletal reorganization ^11– 15^ ( Figure 1). Plasma membrane expansion in developing neurons is facilitated by the insertion of new membrane material via exocytosis ^11, 16^. A classic hypothesis suggests that membrane is added at sites of cell growth, such as the tips of neurites ^17, 18^. An alternative hypothesis suggests that membrane is added distally and membrane material flows laterally toward neurite tips ^16^. Visualization of exocytic hotspots clustered in the soma of neurons indicates that new membrane material is primarily added to the soma ^16^. Although this suggests that lateral membrane diffusion or flow carries membrane material to neurite tips, the dynamics of membrane flow and whether it differs in dendrites and axons are yet to be defined.

In the developing neuron, the cytoskeleton is mainly composed of actin filaments and microtubules (MTs), which each undergo extensive polymerization, depolymerization, and reorganization in response to intrinsic and extrinsic cues. Interactions between actin and MTs mediated by actin and MT crosslinking proteins, such as the spectroplakin family member MACF1 ^19, 20^, help orchestrate cytoskeletal rearrangement and stability ^21^. Nascent branches along the axon and dendrites emerge as actin-rich protrusions known as filopodia. MT invasion of filopodia precedes branch extension and maturation. Classic literature demonstrated that cytoskeletal dynamics and organization differ between axons and dendrites during growth and branching. In the axon, actin forms evenly spaced ring-like structures along the axon shaft ^22^. Actin hotspots observed along axons produce long filamentous actin structures referred to as actin trails ^23, 24^. In contrast, cortical F-actin is described in dendrites and dendritic spines ^14^. MTs in the axon are oriented with their plus-ends outward ^25– 29^. In contrast, dendritic MTs exhibit mixed polarity with 50–90% of their minus-ends oriented away from the soma, dependent upon the organism and neuron type ^25, 26, 30– 32^. The different organization of the cytoskeleton likely underlies differences in cellular architecture and indicates that different regulatory mechanisms for cytoskeletal rearrangement occur in the axon and dendrite.

Local changes in cytoskeletal architecture and plasma membrane addition needed for branching are modulated by activity-dependent and activity-independent mechanisms in a temporally and spatially regulated manner. Extracellular factors such as guidance molecules, neurotrophic factors, and adhesive ligands and their receptors together with intrinsic factors such as organelle position and gene expression initiate and regulate signaling pathways that ultimately converge on the cytoskeleton or exocytic and endocytic machinery to fuel branching ( Figure 1). Here we concentrate on recent advances made in the last three years as an update to classic and recent reviews that illustrate the extensive work that has identified the extracellular and intrinsic factors that regulate neuronal branching ^12, 13, 33– 49^. Although cross-talk between pathways and mechanisms complicates distinction between various factors that regulate branching, we organize recent advances under several broad categories.

## Cytoskeletal changes influence branching

Actin dynamics are critical to axonal and dendritic branching ^12– 14, 33, 37, 42, 45^. Monomeric actin polymerizes to form filamentous actin, which organizes into higher-order structures such as bundles, branched networks, patches, rings, waves, and trails. Multiple isoforms of actin fuel neuronal branching. β-actin is locally translated in distal axons, where it supports axonal growth ^50, 51^. The α-actin isoform is also locally translated and enriched at axonal branch points, where it is critical for collateral branching in motoneurons ^52^. Filopodial protrusions emerge from actin patches preceding branch formation ^53^. In *Caenorhabditis elegans*, the Wnt family of secreted glycoproteins initiates a signaling cascade via endocytosis of its receptor Frizzled that results in actin patch formation and branch initiation sites along the PLM mechanosensory neurons ^54^. Similarly, “actin blobs" mark branch initiation sites along dendrites in *Drosophila* class IV dendritic arborization neurons ^55^. At such branch initiation sites, actin-binding proteins facilitate the reorganization of actin architecture. In *C. elegans*, ** the actin branching Arp2/3 complex is regulated during dendritic arborization by dendritic membrane proteins DMA1 and the claudin protein HPO-30, which form a scaffolding complex for the RacGEF TIAM-1 and WAVE regulatory complex ^56^. Spectrin–actin binding and dynamics are also important for dendritic branching and elongation. An actin-binding domain mutation of β-III-spectrin seen in spinocerebellar ataxia results in tight binding of β-III-spectrin to actin and their accumulation in the soma, leading to reduced dendritic branching in *Drosophila* dendritic arborization sensory neurons ^57^.

MTs help establish and maintain neuronal networks by stabilizing and extending axons, dendrites, and their branches and serving as tracks for motor-based transport ^12, 15, 33, 37, 42, 45, 58^. MTs are composed of 13 laterally associated protofilaments of α-tubulin and β-tubulin heterodimers polymerized in a head-to-tail fashion. Tubulin folding cofactor D (TBCD) facilitates αβ-tubulin heterodimerization ^59^; non-optimal levels of TBCD result in MT disruption and ectopic dendritic arborization ^60^. Filopodia and nascent branches are stabilized by MT entry and destabilized by MT retreat. Growth and catastrophe of MTs are promoted by MT-associated proteins (MAPs) and tubulin post-translational modifications (PTMs) including acetylation, phosphorylation, and polyglutamylation. MAP7 colocalizes with stable acetylated MTs at axon branch points and promotes collateral branching ^61^. MAP7 does not increase branch initiation but rather makes a delayed entry into new branches and is associated with increased MT acetylation, suggesting a potential role for MAP7 in MT stabilization and branch maturation ^61^. β-tubulin phosphorylation by Dyrk1a or its *Drosophila* homolog “minibrain” inhibits tubulin polymerization critical for dendritic branching ^62^. Acetylation of α-tubulin decreases MT dynamics, thereby regulating axonal branching ^63^. *In vitro* and *in vivo* studies suggest that murine headless Myo10 contributes to MT stability and is important for branch formation ^64^, as its deficiency destabilizes MTs and reduces apical dendrite branching. MT destabilization is a mechanism normally associated with the pruning of axonal arbors required to maintain neuronal networks and eliminate synapses. Destabilization of MTs by the severing protein spastin leads to organelle transport deficits and elimination of specific axon branches ^65^. Analogously, downstream of Ube3A/E6AP, MT disruption mediates dendritic arbor shrinkage ^66^. Increased Ube3A/E6AP expression leads to ubiquitination and degradation of X-linked inhibitor of apoptosis proteins, resulting in the activation of caspase3 and MT cleavage.

## Interaction with the extracellular environment influences branching

Adhesions formed by a neuron with other cell types or with the extracellular matrix (ECM) promote or restrict arborization by locally initiating signaling cascades that converge on the cytoskeletal machinery and result in stabilization or destabilization of branches ^67^. Recent studies strengthen these ideas. "Neuritic adhesion complexes" formed between post-synaptic neuroligin in the muscle membrane and pre-synaptic neurexin, syd-1, and liprin-α of adult *Drosophila* pleural muscle motoneurons (PM-Mns) promote axonal arborization ^68^. Live imaging of this process supported a "stick and grow" mechanism ^68^, contrary to the widely accepted "synaptotropic hypothesis", which suggests synapses promote the growth and stabilization of axonal and dendritic arbors. Other cell adhesion molecules that promote arborization include L1-Cell Adhesion Molecule (L1CAM), Contactin-4 (CNTN4), Negr1, and Dystroglycan (DG). L1CAM functions in complex with Ankyrins B and G to positively regulate axonal and dendritic branching ^69^. CNTN4, an axon-associated plasma membrane-anchored cell adhesion molecule, together with amyloid precursor protein (APP), regulates axon branching in the nucleus of the optic tract (NOT) in the accessory optic system ^70^. Negr1 is a GPI-anchored adhesion molecule that regulates branching in a manner dependent on the metalloprotease ADAM10 ^71^. DG similarly positively regulates dendritic arborization in hippocampal neurons via the matrix metalloprotease MMP-9 ^72^, which initiates signal cascades necessary for branch formation. An *in vivo* RNAi screen to identify novel cell surface receptors that regulate dendritic branching in class IV dendritic arborization neurons identified the axon guidance receptor Ret ^73^. Ret interacts with integrins to stabilize dendritic adhesions and maintain even F-actin distribution along the length of dendrites.

Adhesive contacts can also restrict branching. Homophilic γ-PCDH (protocadherin) interactions promote dendritic arborization in the murine cerebral cortex, whereas mismatched homophilic interactions reduce dendritic complexity ^74^. This is contrary to PCDH homophilic interactions leading to repulsion-mediated self-avoidance in retinal starburst amacrine cells (SACs), Purkinje cells, or olfactory sensory neurons ^75– 77^. Neuronal cell shape or variations in signaling pathways in different cell types could explain contrasting outcomes. Enclosure of dendritic regions of *Drosophila* class IV dendritic arborization sensory neurons by epidermal cells, resulting in a loss of integrin-mediated contact with the ECM, reduces branching in enclosed regions ^78^. This is mediated by the epithelial septal junction protein Coracle (Cora) expressed in dendrites and surrounding epidermal cells.

## Cellular organelles central to branching

Neuronal branching increases plasma membrane surface area and cytoplasmic volume. Delivery of organelles such as mitochondria, endoplasmic reticulum (ER), and Golgi outposts to nascent branches facilitates branch growth and maintenance ^42^. Recent studies bolster roles for these organelles in branching. Mitochondria provide ATP for branch formation, which fuels actin dynamics ^79^. Chondroitin sulfate proteoglycans (CSPGs) disrupt the mitochondrial membrane potential and respiratory potential and thus impair actin dynamics and axon branching ^80^. The mitochondrial fission protein GTPase Drp1 is required for dendritic entry of mitochondria. Misregulated expression of Drp1 results in clustering of enlarged mitochondria in the soma and reduced dendritic arborization ^81^. The ER synthesizes proteins and lipids and maintains Ca ^2+^ homeostasis, all necessary for branch formation. Golgi outposts (GOPs) function in post-ER trafficking distal to the soma and are essential for acentrosomal MT assembly in dendrites. Golgi vesicles and recycling endocytic vesicles are transported and delivered to the plasma membrane and potentially provide plasma membrane material for branching ^16, 82^. Indeed, recent mathematical estimations suggest that exocytosis is sufficient to account for plasma membrane expansion in developing neurons ^16^. Several recent studies support this: ablation of genes encoding the GTPase Rab10 and members of the exocyst complex, essential for vesicle trafficking and targeting, results in dendritic arborization defects in *C. elegans* ^83^. Impaired GOP synthesis caused by nuclear polyQ-mediated downregulation of COPII secretory pathway genes results in reduced dendritic branching of *Drosophila* dendritic arborization sensory neurons ^84^. Conversely, local endocytosis triggers thinning and pruning of branches ^85^.

## Transcriptional regulation of branching

The transcriptional control of gene expression has long been appreciated to regulate neuronal morphogenesis, presumably by converging on cytoskeletal or membrane remodeling ^36, 86– 90^. Recent studies continue to further our understanding of the field. The evolutionarily conserved transcription factor (TF) FoxO regulates dendritic branch initiation and stability in *Drosophila* dendritic arborization sensory neurons by modulating MT dynamics and anterograde MT growth ^91^. Sterol regulatory element binding proteins (SREBPs) are TFs critical for the expression of genes necessary for lipid synthesis ^92^. Silencing SREBP expression reduces dendrite length and branching in class IV dendritic arborization neurons in *Drosophila* ^93^. Although glia provide fatty acids for neurite growth ^94, 95^, this recent study suggests that cell-autonomous fatty acid synthesis is also crucial for neuronal morphogenesis. Calcium responsive element binding protein (CREB) is a TF that regulates neuronal morphology in an activity-dependent manner ^96, 97^. A recent study identifies a role for CREB phosphorylation in regulating neuronal architecture in an activity-independent manner during the early stages of neuronal development ^98^. Silencing of the TF Sox5 reduces dendritic branching in *Drosophila* dendritic arborization sensory neurons, possibly by modulating Wnt signaling ^99^. The Pea3 family TFs Etv4 and Etv5 are essential in proper dendritic arborization in hippocampal pyramidal neurons ^100^ potentially downstream of BDNF.

Proteins that alter TF function also affect neuronal branching. The nuclear-nucleolar, intrinsically disordered protein LAPS18-like protein (mLLP) is a cell-permeable protein induced and secreted upon neuronal activity. When added to culture medium, mLLP enhances dendritic arborization by regulating the function of the TF CTCF ^101^. This raises the possibility that the secretion of mLLP is induced by neuronal activity and it is transferred to other neurons to modulate neuronal structure. The activity-dependent kinase cyclin-dependent kinase 5 (CDK5) regulates dendritic arborization. Upon membrane depolarization, CDK5 translocates to the nucleus, where it phosphorylates and inhibits the activity of the repressive TF methyl-CpG-binding protein 2 (MeCP2) ^102^. This leads to the transcriptional activation of *Bdnf* and dendritic arborization.

Chromatin remodeling via DNA methylation and de-methylation, ATP-dependent DNA sliding around the nucleosome, and histone modification modulate gene transcription and neuronal branching patterns ^103– 106^. Knocking out DNA methyltransferase-3 (DNMT3A) in human embryonic stem cells prior to differentiation into motor neurons decreases dendritic arborization ^107^. The Ten-eleven translocation (Tet) family of dioxygenases, which mediate the conversion of 5-methylcytosine (5mC) on DNA to 5-hydroxymethylcytosine (5hmC) ^108^, also modulate branching. 5hmC levels are high at exon start sites of multiple genes upregulated during cerebellar circuitry formation, including axon guidance genes. Tet knockdown inhibits dendritic arborization in cerebellar granule cells, suggesting that Tet regulates 5hmC levels at exon start sites and the transcription of genes regulating dendritic arborization ^109^. ARID1B, a member of the SWI/SNF chromatin remodeling complex, is also critical for regulating dendritic arborization in developing pyramidal neurons ^110^ and thus synaptic function. Truncating mutations or haploinsufficiency of this gene is associated with the developmental disorder Coffin-Siris syndrome, characterized by intellectual disability and speech impairments, autism spectrum disorders, and associated traits such as reduced weight, hydrocephaly, and impaired motor coordination ^111– 116^. The histone deacetylase-4 (HDAC4) and the HDAC-interacting protein ankyrin repeat domain containing protein-11 (ANKRD11) regulate dendritic branching ^117, 118^, indicating that histone acetylation and deacetylation, marks for gene activation and inactivation, respectively, are important players that modulate branching. Both enzymes downregulate the expression of growth factors necessary for morphogenesis. These studies highlight the importance of epigenetic changes in regulating branching morphogenesis and neuronal circuitry.

## Post-transcriptional regulation of branching

Post-transcriptional regulation of gene expression via non-coding microRNAs (miR), alternative splicing, and RNA-binding proteins that alter mRNA conformation are other mechanisms that modulate neuronal branching ^119, 120^. miRs recently shown to regulate branching morphogenesis include *lin-4* miR in *C. elegans* and miR-9, miR-124, miR-128 and miR-16 in the mammalian nervous system ^121– 124^. *lin-4* and its target mRNA encoding the heterochronic TF LIN-14 modulate axonal branching in the PLM neuron in *C. elegans*. miR-9, miR-124, and miR-16 and their target proteins alter dendritic branching by functioning upstream of signaling pathways such as the Akt/GSK3β or MAPK/ERK pathways. Alternative splicing factors such as Caper and RNA-binding Fox1 (Rbfox1) are examples that alternative splicing influences branching ^125, 126^. Caper negatively regulates branching of class IV dendritic arborization neurons in flies by directly or indirectly regulating the expression of over 500 genes, whereas Rbfox1 isoform 2 contributes to dendritic branching by an unknown mechanism. Other RNA-binding proteins that regulate branching include the RNA helicase Moloney leukemia virus 10 (Mov10) ^127^. Cytoplasmic Mov10 possibly renders mRNA, mostly coding for cytoskeletal proteins critical for neuronal morphogenesis, accessible to the RNA-induced silencing complex (RISC) or miRNA-guided cleavage.

mRNA translation also affects arborization and is regulated globally throughout the neuron and locally in the axon or the dendrite ^120, 128^. Density regulated protein (DENR) mediates translation re-initiation after long open reading frames. Overexpression or knockdown of DENR in murine cortical neurons altered spatial dendritic complexity, in particular proximal to the cell body. Furthermore, expression of *de novo* missense mutations identified in two unrelated patients with brain developmental disorder disrupted the function of DENR on mRNA translation re-initiation and the terminal branching of cortical neurons. This suggests DENR’s role in mRNA translation is critical to branching morphogenesis ^129^. The classic translational repressors Nanos (Nos) ^130^ and Pumilio (Pum) repress the expression of the pro-apoptotic gene *head involution defective* ( *hid*) ^131^ and maintain a balance between outgrowth and retraction of dendrites and branches. In contrast, when Hid is upregulated, non-apoptotic caspase activation leads to dendritic arbor pruning. Zipcode binding protein 1 (ZBP1) is a component of the mRNA-binding ribonucleoprotein particle that controls local translation of β-actin mRNA ^132^. ZBP1 binding represses β-actin mRNA translation and ensures proper distribution to sites of local synthesis, where repression is reversed by Y396 phosphorylation on ZBP1. mTORC2, Src, and mRNA binding-dependent S181 phosphorylation of ZBP1 is essential for ZBP1 mobility and distribution along dendrites and regulation of dendritic branching in hippocampal neurons ^133^. An unbiased genome-wide screen identified 55 new candidate mRNA transcripts that localize specifically to dendrites of *Drosophila* class IV dendritic arborization sensory neurons ^134^. Further validation identified 18 genes whose mRNA is transported specifically to dendrites for local translation and regulation of branching. These pathways are likely also relevant *in vivo* in mammals; recent evidence demonstrates that mRNA, and in particular actin mRNA, is transported to docking sites, and subsequent local protein synthesis hotspots in the retinal ganglion axon *in vivo*, and that these sites overlap with branch initiation sites. Thus, sites of local translation may serve as branch initiation sites and regulate branch numbers ^135^.

## Enzymatic activity modulates branching

Various enzymes and PTMs that regulate signaling cascades modulate neuronal branching by directly or indirectly influencing cytoskeletal dynamics and plasma membrane addition ^128, 136– 140^. Recent studies attribute novel roles for numerous enzymes during branching morphogenesis. The methylase coactivator-associated arginine methyltransferase 1 (CARM1) localizes to post-synaptic densities (PSDs) in hippocampal neurons, and CARM1 knockdown leads to exuberant dendritic branching and increased spine density ^141^. CARM1 may function by methylating HuD, an mRNA-binding protein, which prevents HuD from binding and stabilizing mRNA, thereby regulating the expression of proteins directly required for dendrite maturation, such as BDNF. Alternatively, CARM1 may methylate substrates containing an RXR motif and alter protein–protein interactions in the PSD or regulate the expression of downstream proteins such as GTPases and actin regulators ^141^. Methylation via CARM1 downregulates synaptic gene expression and controls neuronal differentiation ^142^, but whether these pathways function in branching has not been shown. PRMT8 is a brain enriched plasma membrane-anchored methyltransferase and phospholipase that promotes dendritic branching. *Prmt8 ^–/–^* mice exhibit reduced dendritic complexity in cerebellar Purkinje cells ^143^. Auto-methylation regulates PRMT8 activity; however, PRMT8 is not known to methylate other substrates proximal to the membrane. PRMT8 does, however, mediate the hydrolysis of phosphatidic choline (PC) to choline and phosphatidic acid (PA). Since PA is a precursor for the synthesis of other phospholipids, PRMT8 may alter the composition and biophysical properties of membranes during axonal and dendritic morphogenesis.

Phosphorylation- and ubiquitination-mediated modulation of signaling plays an important role in neuronal development. For example, activation of the non-receptor tyrosine kinase FAK is required for netrin-dependent cortical axon branching ^144^. In the absence of netrin, the E3 ubiquitin ligase tripartite motif protein 9 (TRIM9) impairs this phosphorylation and activation of FAK via nondegradative ubiquitination of the netrin receptor DCC. Ubiquitination reduces DCC binding to FAK and inhibits subsequent FAK activation. In the presence of netrin-1, TRIM9-mediated ubiquitination of DCC drops, leading to FAK activation, increased exocytosis, and a concomitant increase in axonal branching. TRIM9 negatively regulates axon branching and dendritic arborization in cortical and hippocampal neurons as well ^145, 146^. In contrast to axons, FAK activity negatively regulates dendritic arborization. FAK activation can be inhibited by γ-Pcdh, which binds and inhibits FAK activation ^147^, and thus increases dendritic arborization ^148^. When S922 of γ-Pcdh is phosphorylated by PKC, inhibition of FAK does not occur, ultimately resulting in reduced dendritic arborization in cortical neurons ^149^. Recent studies exploring gene copy number variations such as 16p11.2 microduplication and layer-specific requirements of the ERK/MAPK signaling pathways during neocortical development continue to highlight the importance of the ERK/MAPK signaling pathways in modulating neuronal arborization ^150, 151^.

Dephosphorylation similarly modulates branching, and recent examples demonstrate that localized signaling cascades exert spatial control of branching via dephosphorylation. Straitin-1, a subunit of the S/T phosphatase PP2A, is enriched in striatal neurons, where it negatively regulates dendritic complexity, surprisingly without influencing synaptic connectivity or neuronal activity ^152^. Protein tyrosine phosphatase δ (PTPδ) dephosphorylates and activates Fyn and Src kinases downstream of the repulsive guidance molecule Semaphorin-3A (Sema3A), resulting in increased cortical basal dendritic arborization ^153^. The receptor protein tyrosine phosphatase-69D (RPTP69D) dephosphorylates the cell adhesion molecule DSCAM downstream of Slit ^154^, which promotes the extension of collateral axonal branches.

## Neuronal activity influences branching

Neuronal activity influences branching by regulating calcium activity or inducing transcriptional changes that alter the expression of adhesion molecules, neurotransmitters, growth factors, etc. ^155^. Recent studies support the hypothesis that neuronal activity directly influences branching. When neuronal activity increases, axonal branches in thalamocortical neurons arise from non-synaptic sites ^156^. How activity initiates non-synaptic branch points is not known, but it likely promotes the recruitment of actin-polymerizing factors and other machinery involved in branching to these nascent sites. KIBRA (Kidney and Brain, also known as WWC1) is a somatodendritic phosphoprotein primarily expressed in the hippocampus and cerebral cortex, regions where activity-dependent structural plasticity are critical in memory formation ^157– 160^. KIBRA colocalizes and interacts with GluA1 along dendrites, where it regulates recycling and surface levels of GluA1-containing AMPA receptors and thus activity. Overexpression of KIBRA increases the recycling of GluA1-containing AMPA receptors and also increases the number of branch points along dendrites of murine pyramidal neurons ^161^. Although GluA1 is also known to alter dendritic morphology ^162^, whether KIBRA regulates dendritic branching via GluA1 is unknown.

There is growing evidence that neuronal activity indirectly contributes to branching via the regulation of gene expression. Neuritin is a cell surface-anchored protein upregulated by elevated neuronal activity, such as synchronous firing from an epileptic focus. Neuritin promotes aberrant mossy fiber sprouting of hippocampal granule neurons *in vivo* ^163^, which exacerbates seizure activity. Neuritin overexpression in dissociated hippocampal granule neurons also promotes axonal branching by cooperatively acting with fibroblast growth factor-4 (FGF-4) to recruit FGF receptor (FGFR) and activating the canonical signaling pathway downstream of FGFR. This novel mechanism could contribute to the identification of therapeutic targets for the treatment of epileptic seizures.

## Conclusion

Recent advances in understanding branching morphogenesis have provided insights into activity-dependent and activity-independent extracellular and intrinsic factors that regulate axonal and dendritic branch formation, maintenance, and pruning. The studies reviewed here along with research over the past four decades highlight the complexity of the regulation of neuronal branching. It is evident that cross-talk between pathways operating in parallel regulate branching and fundamental questions remain to be addressed. This includes how signaling pathways and molecules are spatially compartmentalized along the axon and the dendrites, how branches arise from specific points along the axon and dendrite, and how various signaling pathways are coordinated to regulate branching in a temporally and spatially controlled manner.
